# Sialylation and Sulfation of Anionic Glycoconjugates Are Common in the Extracellular Polymeric Substances of Both Aerobic and Anaerobic Granular Sludges

**DOI:** 10.1021/acs.est.2c09586

**Published:** 2023-08-21

**Authors:** Le Min Chen, Stefan de Bruin, Mario Pronk, Diana Z. Sousa, Mark C. M. van Loosdrecht, Yuemei Lin

**Affiliations:** † Department of Biotechnology, Delft University of Technology, Van der Maasweg 9, 2629 HZ Delft, the Netherlands; ‡ Laboratory of Microbiology, Wageningen University & Research, Stippeneng 4, 6708 WE Wageningen, the Netherlands; § Royal HaskoningDHV, Laan 1914 35, Amersfoort 3800 AL, The Netherlands

**Keywords:** EPS, biofilm, size exclusion chromatography, mass spectrometry, nonulosonic acids, glycoconjugates

## Abstract

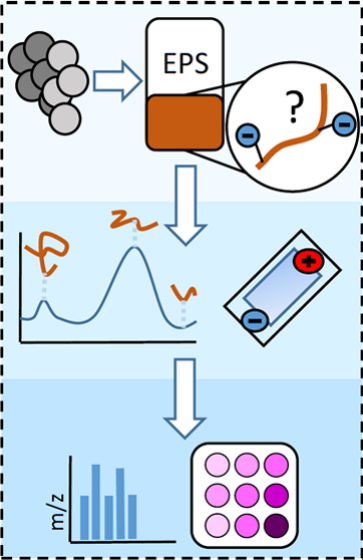

Anaerobic and aerobic granular sludge processes are widely applied in wastewater treatment. In these systems, microorganisms grow in dense aggregates due to the production of extracellular polymeric substances (EPS). This study investigates the sialylation and sulfation of anionic glyconconjugates in anaerobic and aerobic granular sludges collected from full-scale wastewater treatment processes. Size exclusion chromatography revealed a wide molecular weight distribution (3.5 to >5500 kDa) of the alkaline-extracted EPS. The high-molecular weight fraction (>5500 kDa), comprising 16.9–27.4% of EPS, was dominant with glycoconjugates. Mass spectrometry analysis and quantification assays identified nonulosonic acids (NulOs, e.g., bacterial sialic acids) and sulfated groups contributing to the negative charge in all EPS fractions. NulOs were predominantly present in the high-molecular weight fraction (47.2–84.3% of all detected NulOs), while sulfated glycoconjugates were distributed across the molecular weight fractions. Microorganisms, closely related to genera found in the granular sludge communities, contained genes responsible for NulO and sulfate group synthesis or transfer. The similar distribution patterns of sialylation and sulfation of the anionic glycoconjugates in the EPS samples indicate that these two glycoconjugate modifications commonly occur in the EPS of aerobic and anaerobic granular sludges.

## Abstract 2

This study provides insights into the distribution of potential valuable components in the extracellular matrix of the granular sludge, thereby contributing to waste stream valorization.

## Introduction

Microbial granulation is often desired in wastewater treatment processes, as the higher sedimentation velocity of granular sludge allows the ease of biomass separation from treated water.^1,2^ Both aerobic and anaerobic microorganisms can granulate by immobilization in a matrix of self-excreted extracellular polymeric substances (EPS). In both processes, the technology inherently relies on the stability of the granules and the formation of EPS. The importance of negatively charged groups in the EPS for granule stability and for the adsorption of charged substances has been highlighted.^3^ Some studies have focused on negatively charged polysaccharides, for example, bacterial alginate.^4^ However, negatively charged groups can also be found on the glycoconjugates linked to proteins and/or lipids in EPS, e.g., sialic acids and sulfated groups.

Sialic acids are nine-carbon acidic monosaccharides that are mostly detected on the terminal of the glycoconjugate chain in the extracellular matrix of vertebrate cells or pathogenic bacteria.^5^ The most common sialic acids in animal tissue are N-acetylneuraminic acid (NeuAc) and 2-keto-deoxynonulosonic acid (KDN), whereas pseudaminic acid (Pse) and its stereoisomer legionaminic acid (Leg) seem to be exclusive bacterial sialic acids.^6,7^ These are all monosaccharides belonging to a subset of the family of nonulosonic acids (NulOs). Most literature reports on sialic acids have focused on their role in evolution and disease in vertebrates or the interaction between host cells and pathogenic bacteria.^5^ Only very recently, the presence of NulOs in several nonpathogenic microorganisms has been described. NeuAc was found in a diversity of environmental samples and associated with nonpathogenic microbial species.^7−9^ Leg/Pse was predominant in the enrichment of the phosphate-accumulating organism, “*Candidatus* Accumulibacter phosphatis”.^10^ In the S-layer glycoprotein of the Archaea *Halorubrum sp* PV6, Leg was detected and speculated to be important for cell–cell recognition.^11^ It is therefore suggested that glycoconjugates containing these monosaccharides (glycoconjugates with sialylation) may play a role in microbial aggregates, where microbe–microbe interactions occur.

Sulfated groups have been well-studied in mucin and the proteoglycan component in the extracellular matrix of animals, especially in sulfated glycosaminoglycans (GAGs). Sulfated GAGs are highly negatively charged, linear polysaccharide chains, covalently linked to the protein core.^5^ They are involved in distinct functions such as keeping the structural integrity of the extracellular matrix, wound repairing, and cell differentiation in eukaryotes. Like sialic acids, sulfated GAGs have been believed to be produced mostly by pathogenic bacteria. However, recently, the sulfated GAGs have been found in the capsule surrounding the microorganisms and the EPS between the microcolonies in aerobic and anammox granules.^9,12^ In the anaerobic granular sludge, sulfated proteoglycan-like compounds have been reported.^13^

Despite being carbohydrates, sialic acids and sulfated glycoconjugates cannot be detected by frequently used carbohydrate assay, which contributed to so far underestimation of their occurrence, chemical structures, and location in the EPS.^8,12,14−16^ Considering the significant importance of sialylated and sulfated glycoconjugates in the extracellular matrix of animals, further investigation is needed to see if they also are a common factor in the EPS of microbial aggregates beyond pathogenic microorganisms. Research on their chemical structure and secretion will shed light on their specific functionality and evolutionary importance in the extracellular matrix of the biofilm in general.

To investigate the presence of sialylated and sulfated glycoconjugates in the EPS of microbial aggregates and to develop specific methodologies to study them, both aerobic and anaerobic granular sludges were collected from full-scale wastewater bioreactors. The alkaline-extracted EPS of these granular sludges was first fractionated by size exclusion chromatography, and the collected fractions were analyzed in the presence and diversity of the sialic acids and sulfated glycoconjugates. Genes encoding for known enzymes responsible for the synthesis or transfer of sialic acids and sulfate groups were determined by genome database searches on the dominant microorganisms.

## 2 Materials and Methods

### 2.1 Experimental Setup

**Figure 1:**
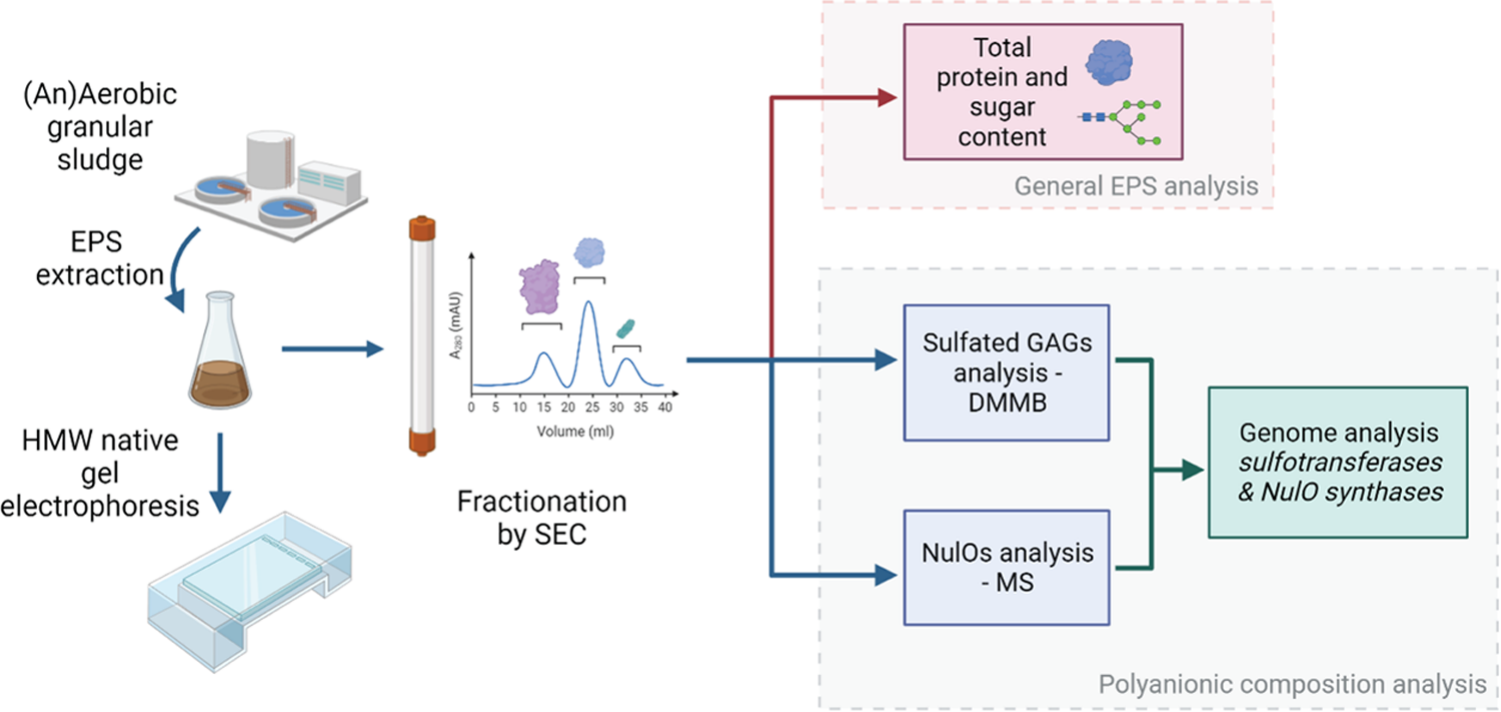
Schematic representation of the workflow of the analysis of the anionic extracellular polymeric substances. Abbreviations: EPS: extracellular polymeric substances, HMW: high molecular weight, SEC: size exclusion chromatography, GAGs: glycosaminoglycans, DMMB: 1,9-dimethyl-methylene blue dye, NulOs: nonulosonic acids, and MS: mass spectrometry.

The analysis of the anionic extracellular polymeric substances extracted from both aerobic and anaerobic granular sludges is summarized in Figure 1.

### 2.2 Granular Sludge and EPS Extraction

The extraction of EPS from both aerobic and anaerobic granules was based on the alkaline heat extraction method described in the previous work.^17−19^ Aerobic granular sludge was collected from two full-scale wastewater treatment plants (Epe and Zutphen) in the Netherlands, which are operated with the Nereda Technology. EPS was extracted by alkaline extraction as explained in detail by Bahgat et al.^20^ for the demonstration plants of Epe (sewage) and Zutphen (dairy). The extraction was performed between pH 9 and 11 by the addition of 25% KOH at 80 °C. The EPS was precipitated afterward by acidification with 30% HCl to pH 2–4. The acid-precipitated EPS was dialyzed to retain polymeric components with a 3.5 kDa-molecular weight cutoff dialysis bag (Snakeskin, Thermo Fisher Scientific), frozen at −80 °C, lyophilized, and stored at room temperature until further analysis.

Anaerobic granular sludge was collected from two full-scale anaerobic granular sludge wastewater treatment systems (treating papermill and brewery wastewaters). The EPS was extracted with alkaline extraction as previously described by Pinel et al.^17^ In short, the EPS was extracted by adding dried biomass to 0.1 M NaOH at 80 °C (10 g/L). The sample was stirred vigorously for 30 min, after which it was cooled and centrifuged at 3300*g* for 30 minutes. The supernatant was dialyzed, lyophilized, and stored following the same procedure as for the EPS from aerobic granular sludge. Detailed information regarding the wastewater treatment process and the type of wastewater is provided in the Supporting Information (Table S1).

### 2.3 Native Agarose Gel Electrophoresis and Staining with Coomassie Blue and Alcian Blue

Native agarose gel electrophoresis was run on a submerged horizontal platform, with the wells positioned in the center of the gel. Lyophilized EPS samples were resolubilized in 50 mM Tris at 2 mg of EPS/mL concentration for 1 hour at 30 °C. Next, 10 μL of the sample was loaded in the wells on a 0.8% agarose gel in 500 mM Tris/HCl, 160 mM boric acid, and 1 M urea, pH 8.5. Electrophoresis was performed with a running buffer (90 mM Tris/HCl, 90 mM boric acid, pH 8.5) at 80 V for 90 min. Proteins carrying a net negative charge migrate toward the anode, whereas proteins carrying a positive charge migrate toward the cathode.^21^ To determine if high-molecular weight proteins could pass the gel, a high-molecular weight marker was used as a ladder (high molecular weight–SDS Calibration kit, Cytiva, Marlborough, MA). The ladder was negatively charged due to the presence of sodium dodecyl sulfate (SDS). The sample position on the gel was revealed using Coomassie blue staining (SimplyBlue Safestain, Invitrogen, Waltham, MA) according to the manufacturer’s instruction and destained in water overnight. To identify the carboxyl groups R-COO^-^ and the sulfated groups R-OSO_3_^–^, staining with Alcian blue was performed at pH 2.5 and pH 1.0, respectively, as described by Boleij et al.^9^ The gel pictures were taken on a ChemiDoc MP imager (Bio-Rad, Hercules, CA).

### 2.4 EPS Fractionation by Size Exclusion Chromatography

EPS samples (10 mg) were solubilized in demineralized water to a concentration of 10 mg EPS/mL, and the pH was adjusted to 10 using NaOH. All solutions were centrifuged and filtered through a 0.45 μm membrane filter before application to the column, to allow the samples to remain dissolved as much as possible.

Size exclusion chromatography (SEC) was performed using a Hiload 16/600 Superose 6 prepacked column (Cytiva Lifesciences, Marlborough, MA) fitted on a Gilson system containing a UV (280 nm) detector. Calibration of the column, upon which the elution volume was determined, was done using a Cytiva high-molecular weight marker set (Cytiva Lifesciences, Marlborough, MA). This consisted of ovalbumin (44 kDa), conalbumin (75 kDa), aldolase (158 kDa), ferritin (440 kDa), thyroglobulin (669 kDa), and blue dextran (2000 kDa). Blue dextran is usually included to determine the void volume, but Superose 6 has a very high fractionation range (fractionation range *M*_r_ ∼ 5 kDa-5000 kDa (globular proteins) and exclusion limit *M*_r_ ∼ 40,000 kDa (globular proteins)); even blue dextran is retained in the column.

Fifteen mL of solubilized EPS samples was run through the column with a flow rate set to 1 mL/min, using a running buffer containing 0.15 M (NaCl) and 0.05 M (glycine) adjusted to pH 10 with NaOH. Five different fractions were chosen based on the retention times of the different proteins in the high-molecular weight marker kit and the extrapolation of the calibration line. EPS fractions were subsequently dialyzed to remove excess salts with a 3.5 kDa-molecular weight cutoff dialysis bag (Snakeskin, ThermoFisher Scientific, Landsmeer), frozen at −80 °C, and lyophilized. The lyophilized samples were stored at room temperature until further analysis.

### 2.5 Characterization of EPS Fractions

#### 2.5.1 Total Protein and Carbohydrate Contents in EPS Fractions

Lyophilized EPS fractions were dissolved in 0.01 M NaOH to 0.5 mg/mL. The total protein content was determined by the BCA protein assay following the manufacturer’s instruction with bovine serum albumin as a standard (Pierce BCA protein assay Kit, Thermo Scientific). Protein absorbance was measured in duplicates at 562 nm using a multimode plate reader (TECAN Infinite M200 PRO, Männedorf, Switzerland). The total carbohydrate content of the EPS solutions, after 2.5 times dilution, was determined by the phenol sulfuric acid method with glucose as a standard.^22^ The carbohydrate absorbance measurements were performed in cuvettes at 490 nm in duplicates with a VIS-spectrophotometer (HACH DR3900, Ames, IA).

#### 2.5.2 Functional Groups of EPS Fractions

Functional group analysis was performed by Fourier transform infrared (FT-IR) spectroscopy on a Spectrum 100 spectrometer (PerkinElmer, Shelton, CT). The spectra of the lyophilized samples were recorded at room temperature over a wavenumber range of 600–4000 cm^–1^ with 16 accumulations and 4 cm^–1^ resolution.^46−49^

#### 2.5.3 Sialic Acid Measurement with Mass Spectrometry

The NulO measurement was performed according to the approach described by Kleikamp et al.^7^ In short, lyophilized EPS fractions were hydrolyzed using 2 M acetic acid for 2 h at 80 °C and dried with a SpeedVac concentrator. The released NulOs were labeled through α-keto acid using DMB (1,2-diamino-4,5-methylenedioxybenzene dihydrochloride) for 2.5 h at 55 °C and analyzed by reverse-phase chromatography–Orbitrap mass spectrometry (QE plus Orbitrap, ThermoFisher Scientific, Bleiswijk, Netherlands). Labeling with other sugars and sugar acids was found to give no DMB derivatives.^23^ To estimate the relative amounts of each type of NulOs, the peak area of 1 μg of KDN was used as a reference signal. The integrated peak areas in the mass spectrometry chromatograms were calculated for each type of sialic acids in each EPS fraction. The peak area was used as a number proportional to the amount of NulOs. The relative amount of each type of sialic acids in each EPS fraction was presented as a ratio to the peak area of 1 μg of KDN for comparison.

#### 2.5.4 Sulfated Glycosaminoglycan Assay

Detection and quantification of sulfated glycosaminoglycans were performed with the Blyscan sulfated glycosaminoglycan assay (Biocolor, Carrickfergus, U.K., the assay range is 0–50 μg/mL and the detection limit is 2.5 μg/mL), according to the manufacturer’s instructions. Samples (2–5 mg) were digested with 1 mL of papain protein digestion solution at 65 °C overnight (Sigma Aldrich, Zwijndrecht, Netherlands). The supernatant was recovered after centrifugation at 13,000*g* for 10 min. 50 μL of sample was then added to 1 mL of 1,9-dimethyl-methylene blue (DMMB) dye reagent. Sulfated GAG-positive components bind and precipitate with DMMB at a low pH (measured pH in the DMMB solution was 1.7). The precipitate was subsequently isolated and resolubilized. The absorbance of the resolubilized solution at 656 nm (TECAN Infinite M200 PRO, Switzerland) indicated the amount of dye that formed a complex with the sulfated glycosaminoglycans. The standard that was included in the kit was bovine tracheal chondroitin 4-sulfate. Due to the low pH, the influence of intracellular components (e.g., DNA) is negligible.^24^ Lastly, the distribution of N-linked and O-linked sulfate in the samples was measured by performing nitrous acid cleavage as per the manufacturer’s instructions prior to sulfated GAG quantification.

### 2.6 BLASTp (Protein Basic Local Search Alignment Tool) Analysis for Nonulosonic Acid Synthases and Sulfotransferases

To identify the 10 most dominant genera of the anaerobic granular sludge community, DNA from sludge samples was extracted using a PowerSoil DNA isolation kit (Qiagen Hilden, Germany) and the V3-V4 regions of the 16S rRNA gene sequenced with primers 341F and 806R.^25^ DNA sequencing was performed at Novogene (Novogene Co., Ltd., China) using the Illumina 51 NovaSeq platform. For aerobic granular sludge, the 10 most dominant genera of the community were selected from the study by Kleikamp et al.^25^ BLASTp from the NCBI website was used to identify the homologous enzymes for the biosynthesis of the NulOs and sulfotransferases in close relative organisms (Table S2) to the most abundant in the anaerobic granular sludge and aerobic granular sludge. The distinct reference protein sequences were taken from bacteria (*Campylobacter jejuni*, *Bacteriodetes thetheiotaomicron*) or archaea (*Halorubrum* sp PV6) known to produce these types of NulOs. Reference proteins for the NulO synthase of Neu5Ac (NeuB), legionaminic acid (LegI), pseudaminic acid (PseI), and 2-keto-3-deoxynonulosonic acid (KDN-9-phosphate) were used, with the corresponding GenBank accession numbers: ERP39285.1, AYD49523, CAL35431, and AAO76821.^11,26^ The distinct reference proteins for sulfotransferase have the accession numbers WP_014336261 and WP_015887312.^9^ Matches with a hit below an *E*-value of 1E-20 were considered significant.

## 3 Results

The EPS was extracted from both anaerobic and aerobic granular sludges with a relatively significant amount, i.e., for the two types of anaerobic granular sludge—treating papermill and brewery wastewater; the extraction yield was 43.3 ± 5.5 and 58.4 ± 0.6%VSS, respectively; for aerobic granular sludge, treating dairy and municipal wastewater, the yield was 22.0 ± 1.7 and 29.0 ± 3.1%VSS, respectively.

### 3.1 EPS Native Agarose Gel Electrophoresis and Staining with Coomassie Blue and Alcian Blue

**Figure 2:**
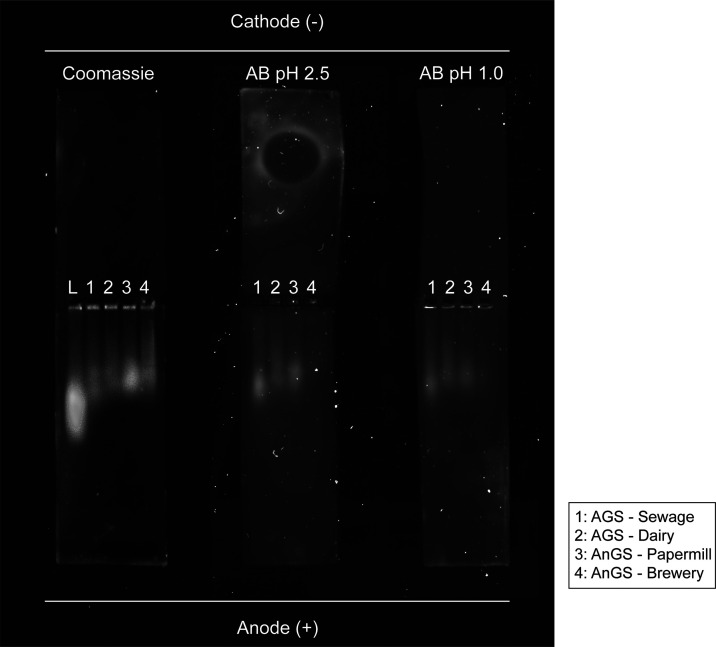
Native gel electrophoresis on agarose stained with Coomassie G-250 (A), with Alcian blue pH 2.5 (B) and pH 1.0 (C) with the crude EPS from aerobic granular sludge-sewage (1), aerobic granular sludge-dairy (2), anaerobic granular sludge-papermill (3), and anaerobic granular sludge-brewery and ladder ranging from 53 to 220 kDa (L). The anode (+) is at the bottom of the gels and the cathode (−) is situated at the top.

The extracted EPS were further analyzed by native agarose gel electrophoresis. Following Coomassie Blue staining (Figure 2A), it was observed that for all EPS samples, a part of the proteins migrated toward the anode (indicative of negatively charged proteins). Another part of the EPS stayed within wells toward the anode, indicating that they may also carry a net negative charge. It is possible that the molecular weight of certain EPS polymers was too high to migrate through the gel.^27^

Alcian blue at pH 2.5 stains both carboxylic and sulfated glycoconjugates, whereas at pH 1.0, it stains only highly negatively charged components, e.g., sulfated glycoconjugates.^9,28^ For each EPS, the protein smear (Figure 2A) and the anionic glycoconjugate smear (Figure 2B,C) almost overlap with each other. In addition, regarding the part that stays within the well, the pattern stained with Alcian blue at pH 1.0 corresponds to the pattern stained with Coomassie Blue as well. All this information implies that the four EPS samples are all dominated by (glyco)proteins, which have carboxylic and sulfated glycoconjugates.

### 3.2 EPS Fractionation and Molecular Weight Distribution

Native agarose gel electrophoresis indicated that the EPS samples are all dominated by (glyco)proteins and have high-molecular weight fractions. To estimate their molecular weight distribution, size exclusion chromatography (SEC) was performed. The detection of proteins’ signal at 280 nm was employed to obtain the chromatogram.^29^ The chromatogram of the EPS samples does not show separate protein peaks but a continuous curve with absorbance at 280 nm (Figure S1). It is noted that glycosylation of proteins leads to a continuous molecular weight distribution rather than a few specific molecular weights, because the level of glycosylation and glycans length can vary for individual proteins.^30,31^ This observation concurs with the glycosylation of proteins by carboxylic and sulfated glycoconjugates observed with the Alcian blue staining in Section 3.2.

The EPS was separated into five fractions: four fractions in the apparent molecular weight range of 5–5500 kDa and one fraction with an apparent molecular weight of >5500 kDa. Overall, for each EPS, the mass of the five apparent molecular weight fractions varies (Table 1). Notably, the highest apparent molecular weight fraction (>5500 kDa) was obtained for every EPS sample and its mass is 16–27% of the mass of the relevant EPS.

**Table 1:** Fractionation Yields for Different EPS Used after Lyophilization (% of Fractionated EPS)^a^

		aerobic granular sludge		anaerobic granular sludge	
fraction #	molecular weight range (kDa)	sewage	dairy	papermill	brewery
1	>5500*	18.8	27.4	17.5	16.9
2	738–5500*	5.6	14.3	7.5	16.2
3	100–738	11.2	15.1	13.5	21.8
4	12–100	19.6	10.4	29.2	14.6
5	3.5–12	24.7	25.2	30.8	24.1
nonsoluble fraction		18.0	7.6	2.4	6.5

a The nonsoluble fraction was not part of the fractionated samples. Samples marked with an asterisk (*) are based on the extrapolation of the calibration line. Actual molecular weights measured would lie between ∼2000 and ∼40,000 kDa.

### 3.3 Characterization of the EPS Fractions

#### 3.3.1 General EPS Characterization: Carbohydrate/Protein Ratio and Functional Group Analysis

**Figure 3:**
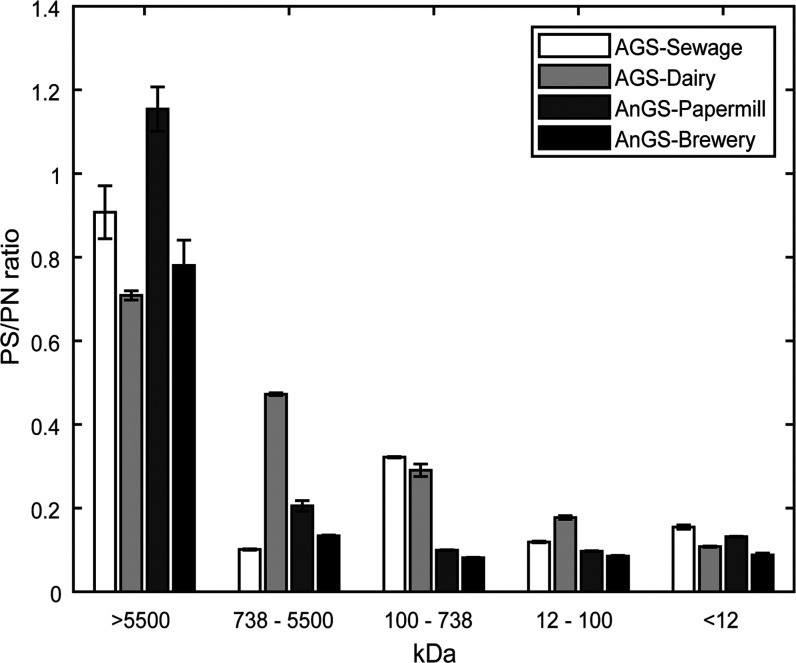
Carbohydrate to protein ratio over the different fractions in aerobic granular sludge-sewage, aerobic granular sludge-dairy, anaerobic granular sludge-papermill, and anaerobic granular sludge-brewery. The carbohydrate content is expressed as glucose equivalents and proteins are expressed as BSA equivalents.

For the fractionated EPS samples, both carbohydrates and proteins were detected in each molecular weight fraction (Figure 3). The sugar to protein ratio (PS/PN ratio) was significantly higher by 2.7–8.6-fold in the highest apparent molecular weight fraction compared to the average of the other fractions. In addition, as the molecular weight decreased, the PS/PN ratio decreased significantly. This indicated that the EPS fractions with an apparent molecular weight of >5500 kDa (16.9–27.4% by weight of EPS) were probably dominated with glycosylated proteins, while the fractions with an apparent molecular weight of <5500 kDa (63.1–81.0% by weight of EPS) were dominated with less or non-glycosylated proteins.

FT-IR spectra were recorded for different EPS fractions to check for the presence of functional groups, e.g., amide groups and C–O–C groups for carbohydrates (Figure S2). In addition to the peaks of proteins (1645 and 1536 cm^–1^) and carbohydrates (1078 cm^–1^), two other peaks, which indicate the presence of sialic acids (1730 cm^–1^) and sulfated esters (1230 cm^–1^), were observed as well.

#### 3.3.2 Nonulosonic Acids

**Figure 4:**
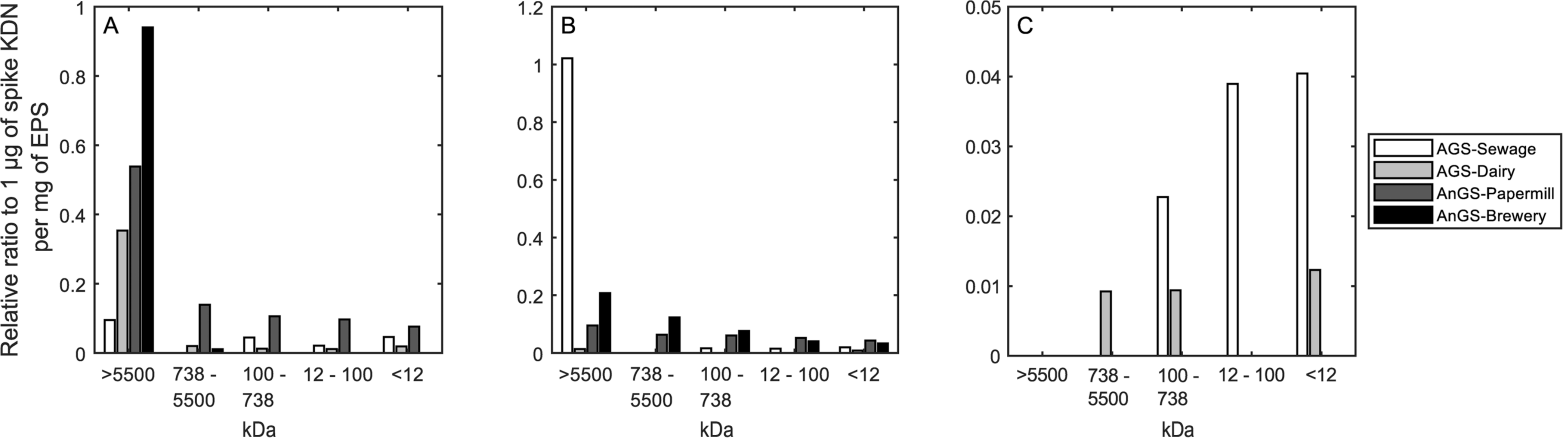
Nonulosonic acids detected in each fraction by MS. The detected NulOs, PseAc2/LegAc2 (A), KDN (B), and NeuAc (C) are expressed as relative ratio of area to spike 1 μg of KDN per mg of EPS in each fraction in aerobic granular sludge-sewage, aerobic granular sludge-dairy, anaerobic granular sludge-brewery, and anaerobic granular sludge-papermill.

To investigate if sialic acids or other types of NulOs are widespread in different EPS fractions, mass spectrometry analysis was performed. As shown in Figure 4, each EPS fraction was sialylated, with diverse types of NulOs and in different amounts. In total, there were three types of NulOs detected: bacterial sialic acids (legionaminic acid (Leg) and/or its stereoisomer pseudaminic acid (Pse)), deaminated neuraminic acid (KDN), and N-acetylneuraminic acid (NeuAc). The most predominant NulO is PseAc2/LegAc2. The highest apparent molecular weight fraction (>5500 kDa) had the highest amount of PseAc2/LegAc2. Especially for the EPS of the two anaerobic granular sludges, PseAc2/LegAc2 was present to a great extent; 75.5% (brewery) and 99.5% (papermill) of their total amount were located at this fraction. In comparison, the EPS of aerobic granular sludge had a slightly lower amount than that of anaerobic granular sludge; about 60.9% (sewage) and 91.3% (dairy) of the total PseAc2/LegAc2 were found in these fractions. The second abundant NulO detected was KDN, except for the EPS from aerobic granular sludge, treating dairy wastewater, which had a low amount of KDN compared to the other EPS. The distribution trend was the same as that of PseAc2/LegAc2: the highest signal of KDN was located at the highest apparent molecular weight fraction (>5500 kDa). Especially for the EPS from aerobic granular sludge (sewage), 95.2% of the detected KDN was at this fraction. In contrast, the relative amount of NeuAc was on average 16-fold lower than the other two types of NulOs. Only the two aerobic granular sludge EPS had NeuAc, which is mainly located at the lower apparent molecular weight fractions (<5500 kDa).

#### 3.3.3 Sulfated Glycosaminoglycans

**Figure 5:**
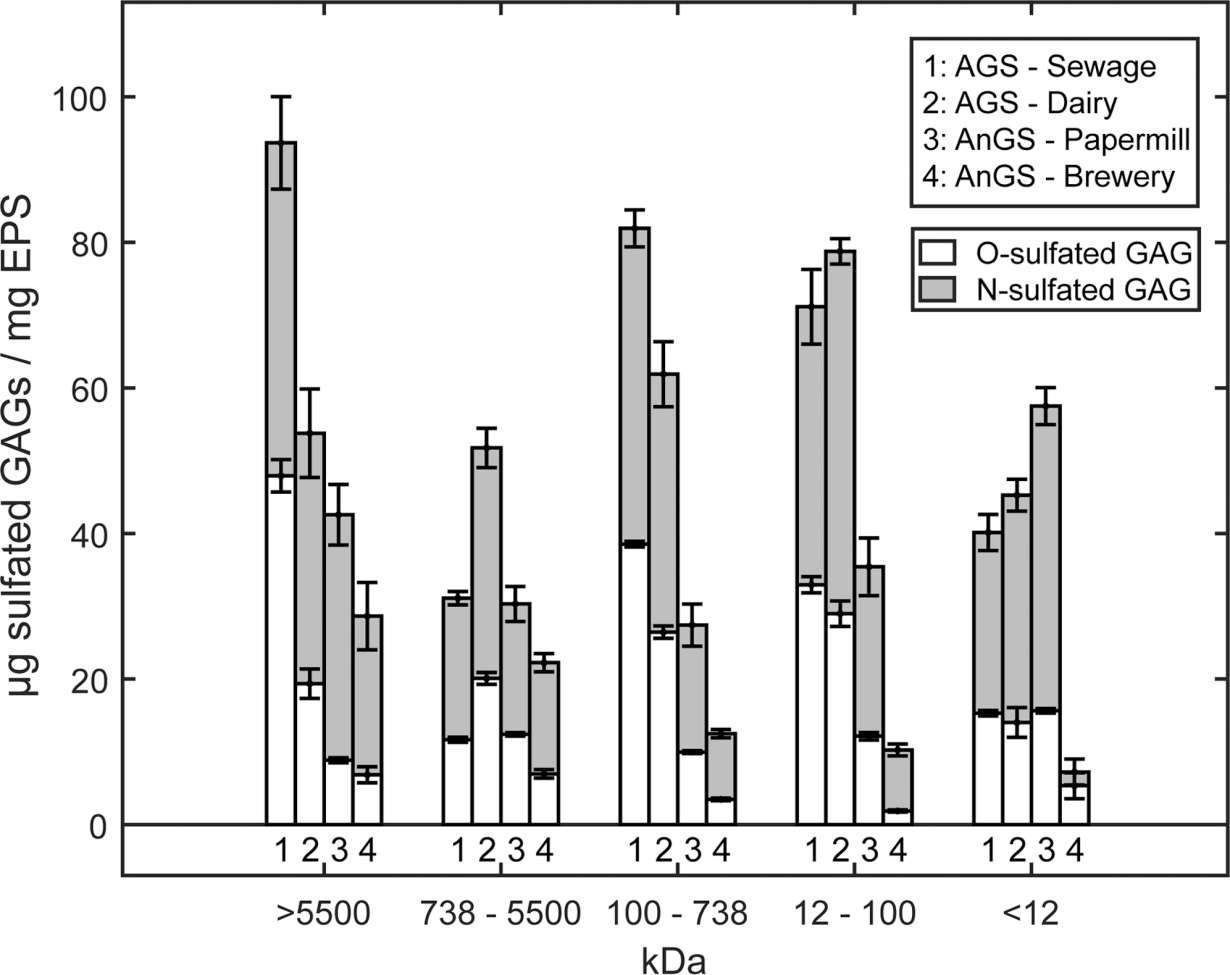
Sulfated glycosaminoglycan concentration (μg of sulfated GAGs/mg of EPS) of O-linked sulfated GAGs (white) or N-linked sulfated GAGs (gray) detected in each fraction in aerobic granular sludge-sewage, aerobic granular sludge-dairy, anaerobic granular sludge-papermill, and anaerobic granular sludge-brewery.

As FT-IR results indicated the possible presence of sulfate esters, the detection and quantification of sulfate esters such as sulfated glycosaminoglycans were performed. Unlike the profile of NulOs, the presence of sulfated GAGs was widely spread across all samples and sample fractions, with no clear trend (Figure 5). The amount ranged from 7.2 ± 0.1 to 93.7 ± 5.7 μg of sulfated GAGs/mg of EPS. On average, across the molecular weight range, the total sulfated GAG content in the aerobic granular sludge EPS was 64.2 ± 2.2 μg of sulfated GAGs/mg of EPS and 55.3 ± 1.9 μg of sulfated GAGs/mg of EPS, for granular sludge from sewage and dairy, respectively. In the anaerobic granular sludge EPS, 42.1 ± 1.6 μg of sulfated GAGs /mg of EPS and 15.4 ± 0.9 μg of sulfated GAGs/mg of EPS, for granular sludge from the papermill and brewery, respectively. In addition to sulfated GAGs, O-linked sulfated GAGs and N-linked sulfated GAGs were determined separately. In the case of aerobic granular sludge, the average weighted percentage of O-linked sulfated GAGs was found to be 46.1 ± 8.6 and 36.6 ± 7.0% in the fractions, for sewage and dairy, respectively, while for anaerobic granular sludge, 29.4 ± 5.8 and 31.9 ± 8.2% O-linked sulfated GAGs were found for the papermill and brewery, respectively. Overall, the percentage of O-linked sulfated GAGs was lower than the N-linked sulfated GAGs.

### 3.4 Genome Analysis of Sulfotransferases and Nonulosonic Acid Synthases

**Figure 6:**
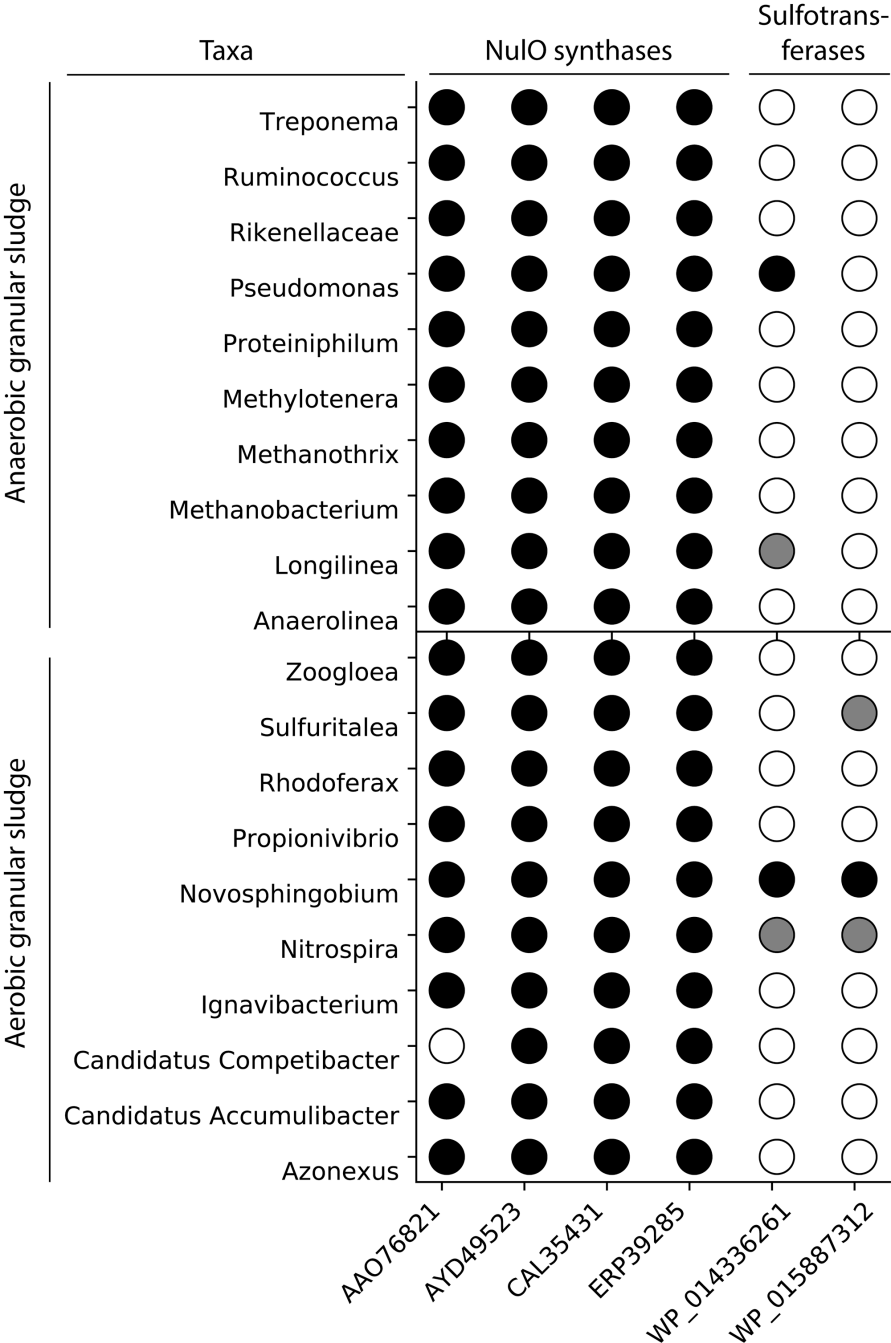
BLASTp analysis of sulfotransferases and the nonulosonic acid synthases over the top 10 most abundant genera in the microbial community of anaerobic granular sludge and aerobic granular sludge. Hits are indicated as a black circle, when the BLASTp analysis showed a match with an *E*-value lower than 1E-20. No hits are indicated as a white circle. Lowering the threshold to 5E-2 revealed more distant hits for sulfotransferases, indicated as a gray circle.

To further evaluate the production potential of NulOs and sulfated polymers, BLASTp was performed with key proteins for the formation of these compounds on representative organisms of the top ten most abundant genera in the microbiome of aerobic granular sludge and anaerobic granular sludge (Figure 6). Sulfotransferases, the enzyme which transfers sulfo groups onto polysaccharides, and NulO synthases, the enzyme responsible for the condensation of a 6-carbon sugar with the 3-carbon phosphoenolpyruvate to generate the 9-carbon Leg, Pse, Neu5Ac, and KDN, were used as a reference. Most of the genomes from the mined microorganisms contain homologous genes for the NulO biosynthesis of either Neu5Ac, Leg, or Pse, implying that these organisms can synthesize NulOs. Hits that matched with the selected NulO synthases were mainly annotated as pseudaminic acid synthase or *N*-acetylneuraminic acid synthase in the community of aerobic granular sludge and anaerobic granular sludge. A few hits were found for genes annotated with *N*,*N*′-diacetyllegionaminic acid synthase (LegI). Differentiation between Neu5Ac and KDN synthases cannot be made due to the way the genes are annotated in the database.

Hits for the sulfotransferases were less abundant. Only few organisms related to representative organisms in the anaerobic granular sludge and aerobic granular sludge microbiome showed positive hits for sulfotransferases. However, when lowering the threshold for the BLASTp search to 5E-2, more organisms showed hits with genes annotated with sulfotransferases. This suggests that the genes encoding for sulfotransferases might have a more distant relation to the reference protein than what is reported for NulOs.

## 4 Discussion

### 4.1 Sialylation and Sulfation of Anionic Glycoconjugates Are Common in the Extracellular Polymeric Substances of Both Aerobic and Anaerobic Granular Sludges

In the current research, the EPS of anaerobic and aerobic granular sludges collected from two different types of wastewater treatment systems were extracted. The sialylation and sulfation of anionic glycoconjugates in the EPS were investigated. Three types of NulOs were detected, with significant differences in their amount and location: both bacterial sialic acids (PseAc2/LegAc2) and KDN were much more abundant than NeuAc (with the relative amount almost 30 times of NeuAc); the majority of both PseAc2/LegAc2 and KDN were located in the highest molecular weight fraction, while NeuAc was only present in the lower molecular weight fractions of the EPS recovered from the aerobic granular sludge. Different from NulOs, the sulfated GAGs were equally distributed over every molecular weight fraction.

The presence of sulfated glycosaminoglycans and NulOs in the aerobic granular sludge, anaerobic granular sludge, and anammox granular sludge was reported recently.^8,9,12,13^ Each study focused on either one specific glycoconjugate or one specific sludge. In comparison, in the current research, granular sludges from different waste streams, enriched with different microbial communities (e.g., aerobic and anaerobic microorganisms) and operated under different conditions (e.g., temperature and pH), were investigated. The EPS extraction methods were not identical for the different sludge samples: although both extraction methods are based on harsh alkaline extraction, they varied in scale (i.e., full-scale and lab-scale extraction), the type of the base (i.e., KOH and NaOH), and the subsequent recovery of the solubilized EPS (acidic precipitation and dialysis). Despite all these differences, it was still observed that NulOs and sulfated groups were present in the EPS samples with similar trends in their abundance and their location at different molecular weight fractions.

Based on the current work and the previous reports, a conclusion can be drawn that sialylation and sulfation of anionic glycoconjugates are widely distributed in the extracellular polymeric substances of granular sludge and could be a common phenomenon in environmental biofilms in general. This provides the support that the function of sialylated and sulfated glycoconjugates produced by the microorganisms is not just limited to being a camouflage to avoid the detection of the host immune system, as suggested for pathogenic bacteria, but could be involved in structural components of the granule.

One could speculate on potential functions of sialylated and sulfated glycoconjugates by looking at the role of analogous compounds in animal tissue. Glycosaminoglycans are well-defined polyanionic compounds in the extracellular matrix of animals. These high-molecular weight compounds are found widely distributed in the connective tissue, creating a highly porous and hydrophilic hydrogel structure.^32^ Furthermore, sialic acids have also been well described in vertebrate cells and are involved in hydration, protein stabilization, and cell–cell interactions, due to their negative charge.^33^ The mass of the highest molecular weight fraction (>5500 kDa) comprised 16–27% of the total EPS and was the most glycosylated fraction with a measured carbohydrate content of 35–58%. Interestingly, a similar characterization was reported for mucin. Mucins are space-filling large molecular weight glycoproteins (20–20,000 kDa) with 50–90% carbohydrate content. The mucin glycoproteins may be sialylated and/or sulfated.^34,35^ The carbohydrate part is largely involved in the mucin properties, such as hydration, binding of ions and water, and protease inhibitors.^35^ It can be speculated that the function of the highest molecular weight EPS fraction might be similar. The exact role of these glycoconjugates inside the extracellular matrix of the environmental biofilm is an interesting topic for future investigation.

### 4.2 Separation and Enrichment of the Sialylated and Sulfated Glycoconjugates by Size Exclusion Chromatography with a High-Molecular Weight Column

The separation and enrichment of glycoconjugates of the extracted EPS aid in further analysis regarding the exact linkage of monomers and chemical structure of these glycoconjugates. This is necessary for a better understanding of the function and production of glycoconjugates in the biofilm. Since most sialic acids are on the highest molecular weight fraction (>5500 kDa), which is heavily glycosylated and sulfated, enrichment can be achieved at the same time of separation. In this respect, applying a SEC column which can separate large molecular weight polymers is necessary and important. It is noted that most fractionation studies done with microbial EPS seldom consider protein glycosylation and use columns that can separate molecules up to 670 kDa.^36−38^ Frequently, the fraction with a molecular weight of >2000 kDa is not analyzed since it exceeds the void volume of the column. If the result of the current research is considered, ignoring this fraction leads to the loss of almost 1/3 of the extracted EPS, not to mention the consequence that most of the sialylated EPS could never be collected and studied.

High-molecular mass biopolymers (molecular weight of >2000 kDa) are common in nature and are found for instance in the extracellular matrix of vertebrates. Aggrecan, a highly glycosylated and sulfated proteoglycan in the articular cartilage, can have a molecular mass of up to 2000 kDa.^39^ It has been speculated in the literature that the bacterial EPS is chemically similar to mucin.^40^ Mucin, which is both a sialylated and sulfated glycoprotein complex, has a molecular mass of 20–20,000 kDa.^34,35^ Sialylated and/or sulfated glycoconjugates can tremendously increase the molecular mass of proteins.

### 4.3 Bottlenecks in the Study of Sialylated and Sulfated Glycoconjugates in the EPS

Although in the current research, by chemical analysis, both sialylated and sulfated glycoconjugates are found widely spread in all the EPS samples, in the genome analysis of sulfotransferases, very few microorganisms from the most abundant ones in both the aerobic granular sludge and anaerobic granular sludge showed positive hits for sulfated glycosaminoglycan sulfotransferases. The reason could be that sulfotransferases were searched in close relatives, which do not have this specific gene. Analyzing the metagenome of these samples would improve the estimation of the sulfated glycoconjugate production potential. However, analysis of glycoconjugate production from metagenome data is not trivial. This is due to the fact that metabolic pathways of sulfated GAG production in bacteria are not well known. The known reference sulfotransferases may not be the enzymes involved in the formation of sulfated glycoconjugates in the EPS. Thus, knowing the exact chemical structure of the sulfated glycoconjugates can aid in finding sulfated polymer production pathways. At present, the methodologies used to study the sulfated glycoconjugates in the EPS depend on dye-spectrometric methods, i.e., visualization by Alcian blue staining and heparin red staining and quantification by DMMB staining.^9,12,41,42^ These methods are useful for indicating the presence of sulfated GAGs or other sulfated polymers but have difficulty in distinguishing between different types of sulfated polymers. Sensitive methods such as, e.g., MS/MS or liquid chromatography (LC)-fluorescence and LC-mass spectrometry (MS), could be used to distinguish between the types of sulfated polymers.^43^ As the wide range of molecular size and type of EPS may increase the complexity, the separation of different molecular weight EPS fractions could help decrease the complexity and thereby improve the identification of the sulfation patterns.

Separating the EPS by SEC would also help further determine the exact molecular location of bacterial sialic acids and KDN. Determining the saccharide sequence that the sialic acids are attached to would reveal more information about the structure–function relationship to understand their role in the EPS. Unfortunately, no sequencing tool such as that existing in proteomics or genomics is available to date for glycoconjugates, since glycoconjugates are far more complex and diverse than proteins and nucleic acids.^44^ In addition, the diversity of NulO types and modification increase the complexity even more. Altogether, studying NulOs from an environmental sample is challenging. Therefore, to better understand the production and diversity of NulOs in EPS, the lectin array or glycoengineering methods can provide novel insights.^45^

## Supporting Information Available

Information on the wastewater treatment process where samples were collected, NCBI tax id and respective number of genomes used in the genome analysis, chromatograms of size exclusion chromatography runs, yields of the subfractions and nonsoluble fraction of size exclusion chromatography run, absorbance band annotation, and FT-IR absorbance spectra for the EPS of different molecular weight fractions (PDF)
