# Supplementary material for: Sialylation and Sulfation of Anionic Glycoconjugates Are Common in the Extracellular Polymeric Substances of Both Aerobic and Anaerobic Granular Sludges
Source: Environ Sci Technol. 2023 Aug 21;57(35):13217–25. doi: 10.1021/acs.est.2c09586 (PMC10483923; doi:10.1021/acs.est.2c09586)
Supplement: Supplementary file 1 — es2c09586_si_001.pdf [file es2c09586_si_001.pdf]

**Sialylation and sulfation of anionic glycoconjugates are common in the extracellular polymeric substances of both aerobic and anaerobic granular sludge**

**Lemin Chen[1†], Stefan de Bruin[1†*], Mario Pronk[1,3], Diana Z. Sousa[2], Mark C.M. van Loosdrecht[1], Yuemei Lin[1]**

[1] Department of Biotechnology, Delft University of Technology, Van der Maasweg 9, 2629 HZ Delft, the Netherlands

[2] Laboratory of Microbiology, Wageningen University & Research, Stippeneng 4, 6708 WE Wageningen, the Netherlands

[3] Royal HaskoningDHV, Laan 1914 35, Amersfoort 3800 AL, The Netherlands

[†]Both authors contributed equally

[*]Corresponding author: s.debruin@tudelft.nl

## Supplemental Information

Pages: S1 – S8

List of figures:

**Supplemental Figure S1.** Chromatogram of SEC runs at 280 nm of the extracted EPS of AGS-Sewage (A), AGS-Dairy (B), AnGS-Papermill (C) and AnGS-Brewery (D).

**Supplementary Figure S2.** FTIR Spectra of the EPS fractions and subfractions of AGS-Sewage (A), AGS-Dairy (B), AnGS-Papermill (C) and AnGS-Brewery (D).

List of tables:

**Supplemental Table S1.** Information of the aerobic and anaerobic wastewater treatment process where the sludge was collected.

**Supplemental Table S2.** NCBI tax id and their respective number of genomes of the microorganisms used in the genome analysis.

**Supplemental Table S3.** Subfractions yields for different EPS used after lyophilization (% of fractionated EPS). The non-soluble fraction was not part of the fractionated samples.

**Supplemental Table S4.** Functional group assignment of absorbance in specific wavenumbers.

**Supplemental Table S1.** Information of the aerobic and anaerobic wastewater treatment process where the sludge was collected.

| | AGS-Sewage | AGS-Dairy | AnAGS-Brw | AnAGS-Pap |
|---|---|---|---|---|
| wastewater treatment process | Nereda© Technology* | Nereda© Technology* | UASB | IC |
| Wastewater type | 75% municipal<br>25% slaughterhouse | Industrial wastewater from dairy factory | Industrial wastewater from brewery | Industrial wastewater from paper industry (recycle and kraft) |

*Nereda® is a registered trademark for an aerobic granular sludge technology owned by Royal HaskoningDHV (Pronk et al (2015). Full scale performance of the aerobic granular sludge process for sewage treatment. Water Research, 84, 207–217. https://doi.org/10.1016/j.watres.2015.07.011)

**Supplementary Table S2.** NCBI tax id and their respective number of genomes of the microorganisms used in the genome analysis.

| GS type | Name organism | NCBI:txid | Number of genomes |
|---|---|---|---|
| AnGS | Treponema | 157 | 1240 |
| | Ruminococcus | 1263 | 2,149 |
| | Rikenellaceae | 171550 | 1,620 |
| | Pseudomonas | 286 | 26,150 |
| | Proteiniphilum | 294702 | 62 |
| | Methylotenera | 359407 | 80 |
| | Methanothrix | 2222 | 113 |
| | Methanobacterium | 2160 | 120 |
| | Longilinea | 475961 | 5 |
| | Anaerolinea | 233189 | 49 |
| AGS | Zoogloea | 349 | 29 |
| | Sulfuritalea | 1054211 | 32 |
| | Rhodoferax | 28065 | 146 |
| | Propionivibrio | 83766 | 28 |
| | Novosphingobium | 165696 | 246 |
| | Nitrospira | 1234 | 299 |
| | Ignavibacterium | 795750 | 33 |
| | Candidatus Competibacter | 221279 | 6 |
| | Candidatus Accumulibacter | 327159 | 82 |
| | Azonexus | 146936 | 20 |

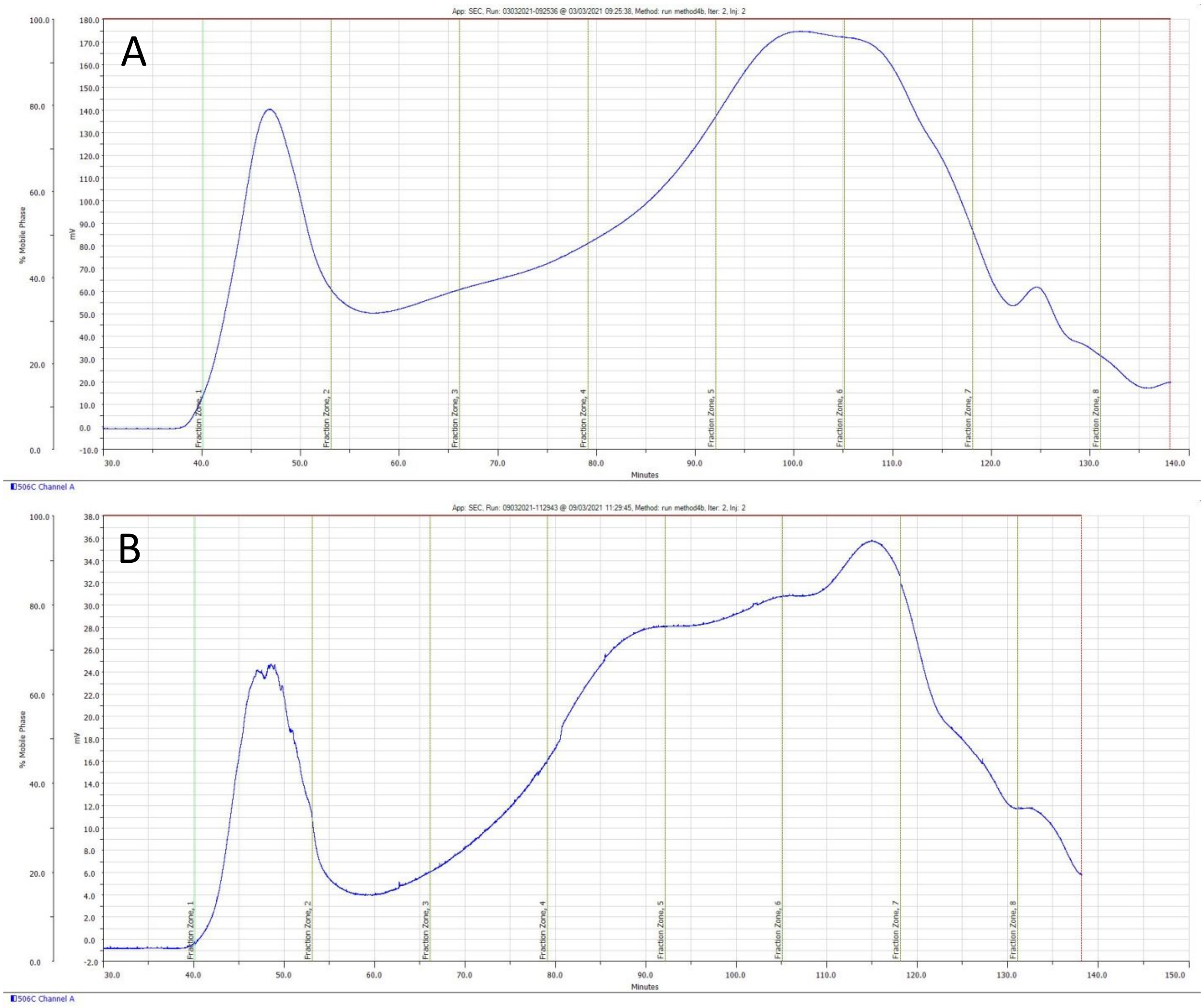
A
App: SEC, Run: 03032021-092536 @ 03/03/2021 09:25:38, Method: run method4b, Iter: 2, Inj: 2
% Mobile Phase
mV
Minutes
Fraction Zone, 1
Fraction Zone, 2
Fraction Zone, 3
Fraction Zone, 4
Fraction Zone, 5
Fraction Zone, 6
Fraction Zone, 7
Fraction Zone, 8
506C Channel A
B
App: SEC, Run: 09032021-112943 @ 09/03/2021 11:29:45, Method: run method4b, Iter: 2, Inj: 2
% Mobile Phase
mV
Minutes
Fraction Zone, 1
Fraction Zone, 2
Fraction Zone, 3
Fraction Zone, 4
Fraction Zone, 5
Fraction Zone, 6
Fraction Zone, 7
Fraction Zone, 8
506C Channel A

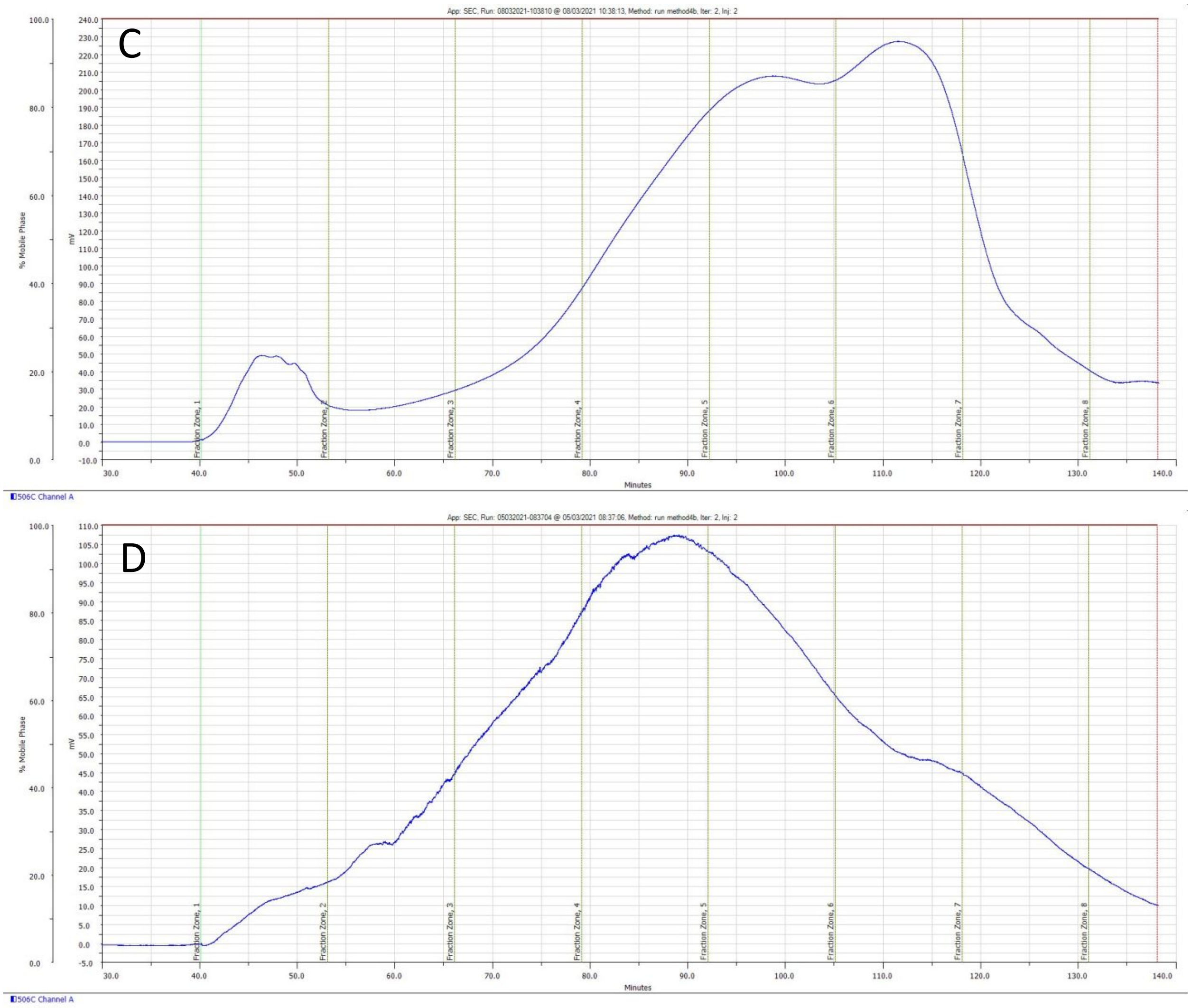


**Supplemental Figure S1.** Chromatogram of SEC runs at 280 nm of the extracted EPS of AGS-Sewage (A), AGS-Dairy (B), AnGS-Papermill (C) and AnGS-Brewery (D)

**Supplemental Table S3.** Subfractions yields for different EPS used after lyophilization (% of fractionated EPS). The non-soluble fraction was not part of the fractionated samples.

| fraction # | MW range (kDa) | AGS-Sewage (% of fractionated EPS) | AGS-Dairy (% of fractionated EPS) | AnGS-Papermill (% of fractionated EPS) | AnGS-Brewery(% of fractionated EPS) |
|---|---|---|---|---|---|
| 1 | >15,000 | 11.8 | 14.9 | 10.1 | 6.7 |
| 2 | 5,500–15,000 | 7.0 | 12.5 | 7.4 | 10.2 |
| 3 | 738 – 5,500 | 5.6 | 14.3 | 7.5 | 16.2 |
| 4 | 100 - 738 | 11.2 | 15.1 | 13.5 | 21.8 |
| 5 | 12 - 100 | 19.6 | 10.4 | 29.2 | 14.6 |
| 6 | 5 - 12 | 18.6 | 15.5 | 22.4 | 14.3 |
| 7 | 3 - 5 | 8.1 | 9.7 | 8.4 | 9.8 |
| Non soluble fraction | | 18.0 | 7.6 | 2.4 | 6.5 |

**Supplemental Table S4.** Functional group assignment of absorbance in specific wavenumbers.

| Wavenumber | Functional group assignment | reference |
|---|---|---|
| 1730 cm-1 | v(C=O)OH stretch sialic acid | 8 |
| 1645 cm-1 | Protein Amide I | 46, 47 |
| 1536 cm-1 | Protein Amide II | 46, 47 |
| 1230 cm-1 | $v_{as}$ S=O stretch of sulfate ester | 48 |
| 1078 cm-1 | -CO (β-glycosidic) carbohydrates | 49 |
| 1025 cm-1 | -CO (α-glycosidic) carbohydrates | 49 |

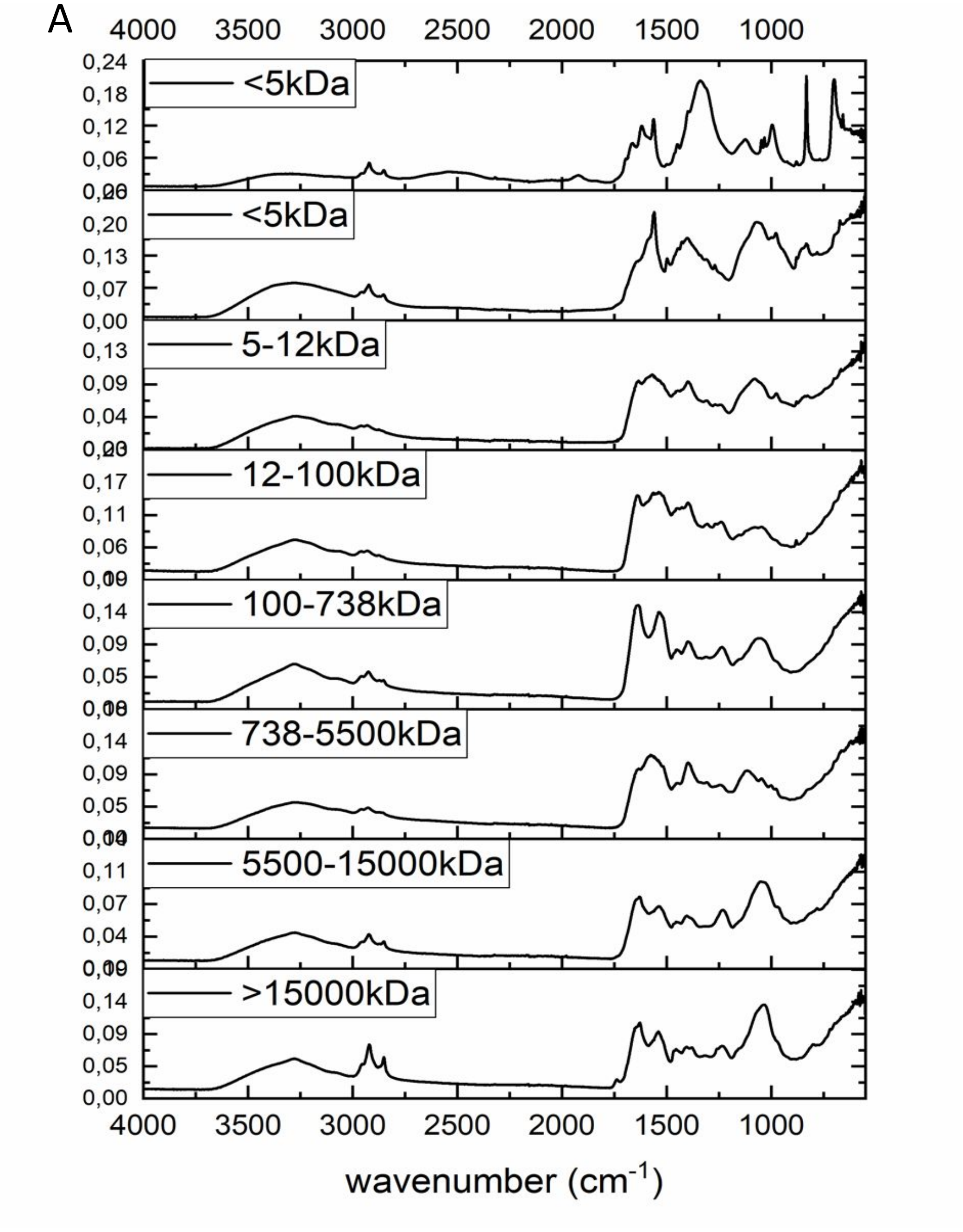
A
<5kDa
<5kDa
5-12kDa
12-100kDa
100-738kDa
738-5500kDa
5500-15000kDa
>15000kDa
wavenumber (cm-1)

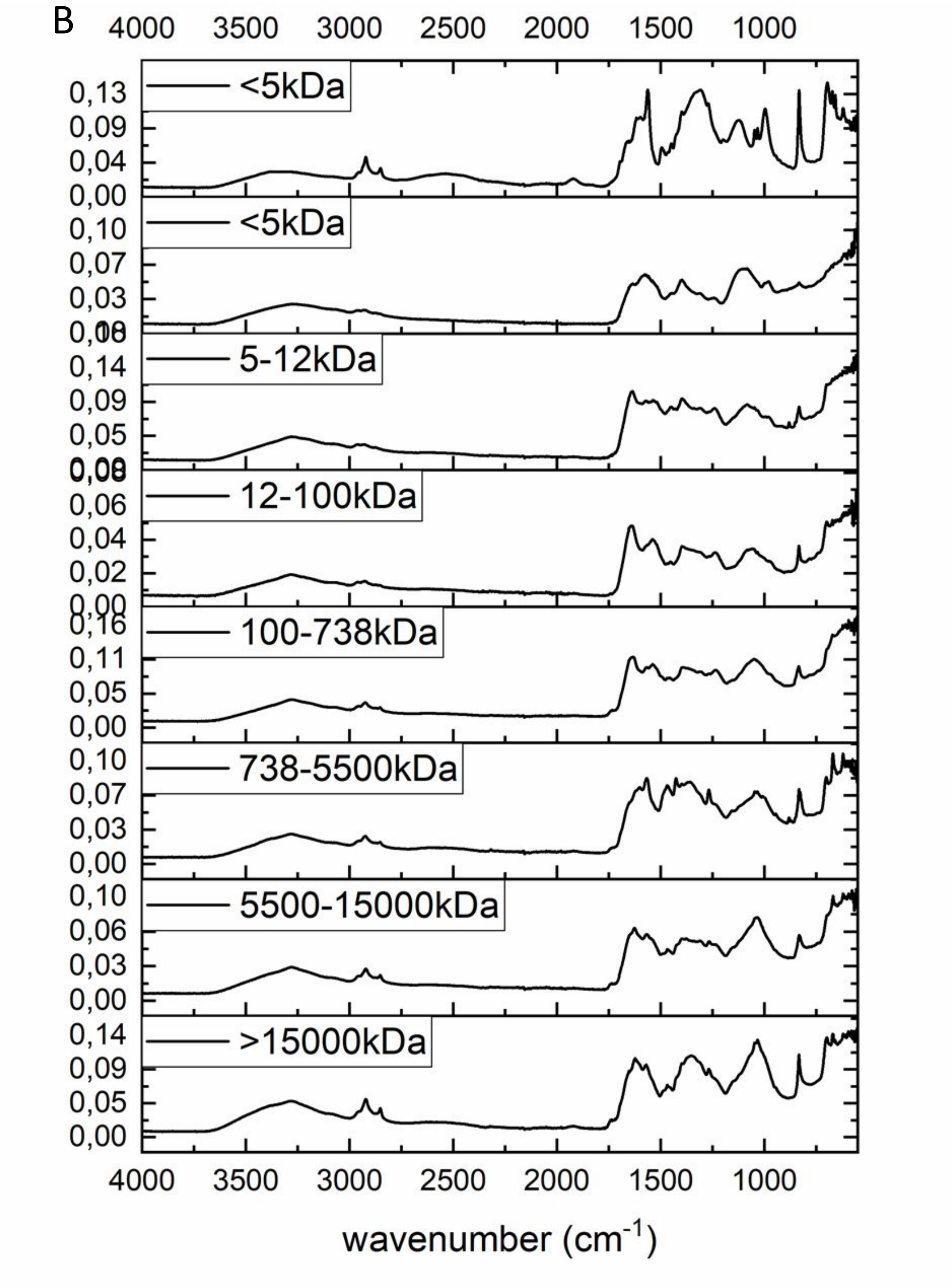

B
<5kDa
<5kDa
5-12kDa
12-100kDa
100-738kDa
738-5500kDa
5500-15000kDa
>15000kDa
4000
3500
3000
2500
2000
1500
1000
wavenumber ($cm^{-1}$)

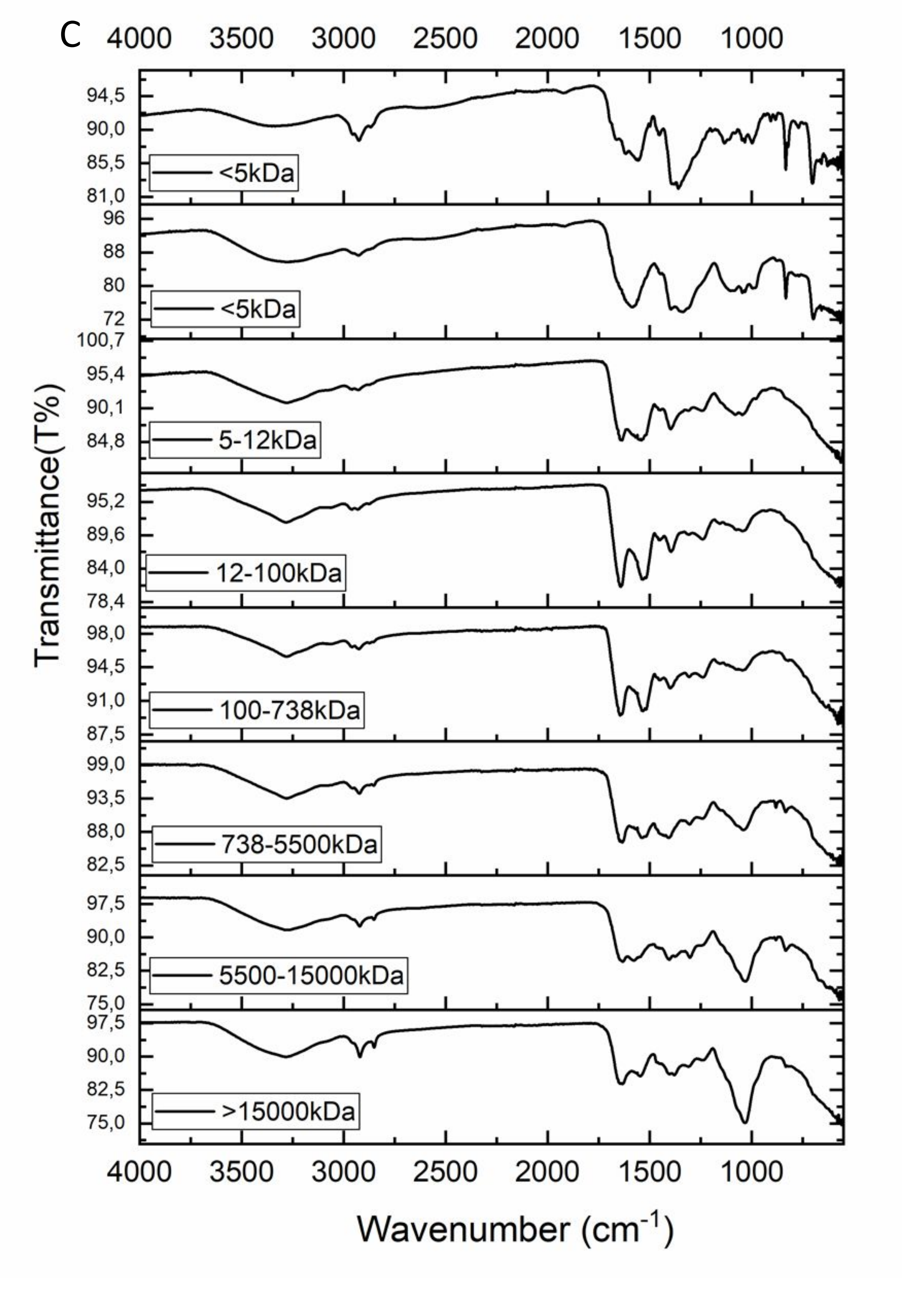
C
4000
3500
3000
2500
2000
1500
1000
Transmittance(T%)
Wavenumber (cm-1)
94,5
90,0
85,5
81,0
<5kDa
96
88
80
72
<5kDa
100,7
95,4
90,1
84,8
5-12kDa
95,2
89,6
84,0
78,4
12-100kDa
98,0
94,5
91,0
87,5
100-738kDa
99,0
93,5
88,0
82,5
738-5500kDa
97,5
90,0
82,5
75,0
5500-15000kDa
97,5
90,0
82,5
75,0
>15000kDa

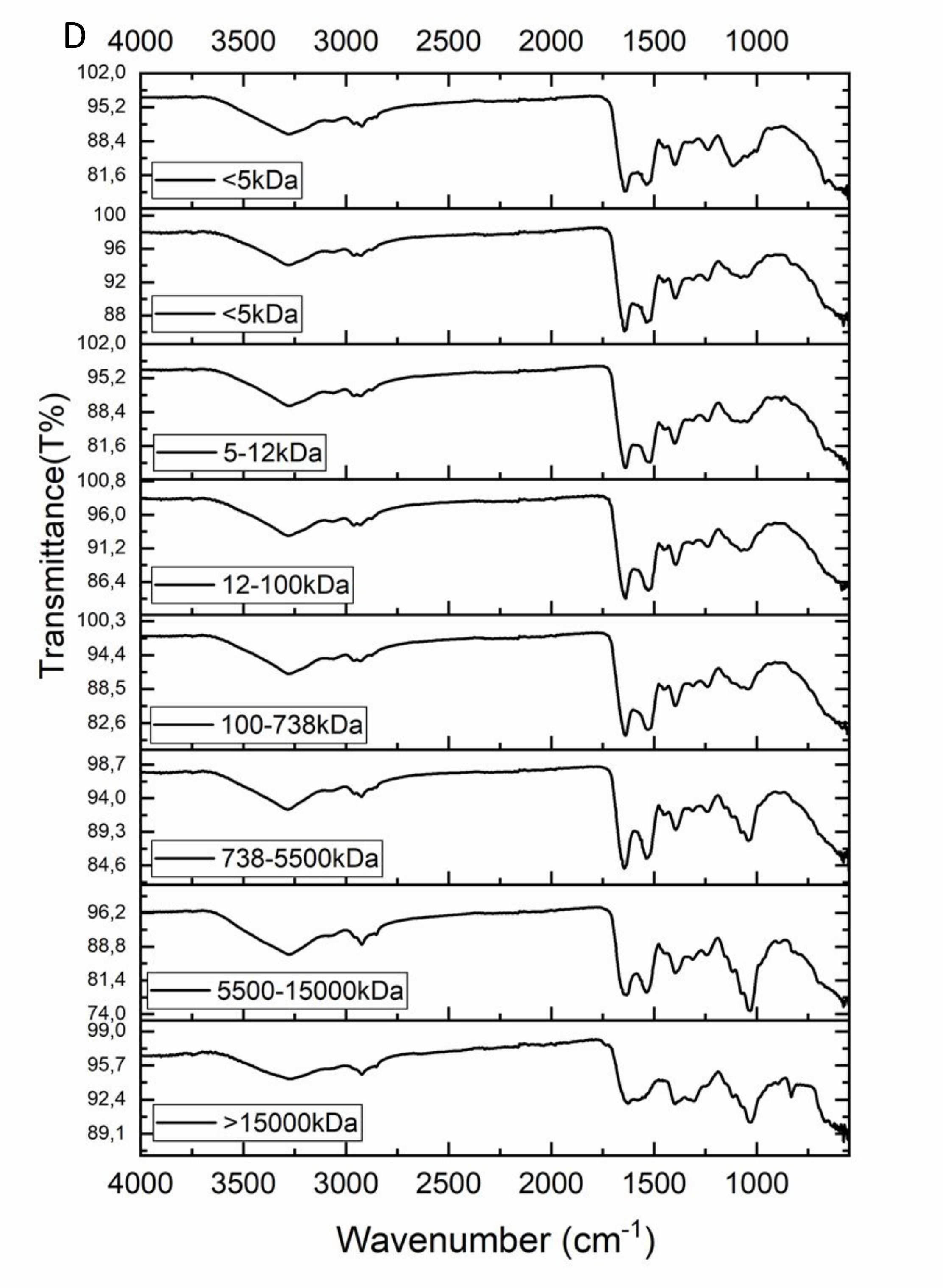


**Supplementary Figure S2.** FTIR Spectra of the EPS fractions and subfractions of AGS-Sewage (A), AGS-Dairy (B), AnGS-Papermill (C) and AnGS-Brewery (D). The reference wavelengths were identified to its according functional group (Table S4).
